# Frailty and Anemia in Geriatric Care: A Retrospective Cohort Study

**DOI:** 10.1097/AJN.0000000000000173

**Published:** 2025-10-23

**Authors:** Hongyan Wang, Ye Qiu, Ning Li, Xiaohan Zhou, Dongying Chen

**Affiliations:** **Hongyan Wang, Ye Qiu, Ning Li, Xiaohan Zhou,** and **Dongying Chen** are all RNs at The First Affiliated Hospital of Soochow University, Suzhou, Jiangsu, China. Contact author: Dongying Chen, jxf552@sina.com. The authors have disclosed no potential conflicts of interest, financial or otherwise.

**Keywords:** anemia, frailty, geriatric, nursing, older adults, treatment

## Abstract

**Background:**

Frailty is a prevalent geriatric syndrome that markedly diminishes the functional capacity and overall quality of life of older adults. Anemia, a common comorbidity in geriatric populations, has been associated with adverse health outcomes. Both frailty and anemia are highly prevalent among geriatric inpatients.

**Purpose:**

This study aims to explore the relationship between frailty and anemia among older hospitalized patients, thereby contributing to the development of evidence-based strategies for identifying and managing the care of these vulnerable patients.

**Methods:**

We conducted a retrospective cohort study involving patients 65 years of age and older admitted to our hospital's geriatric department between October 2022 and August 2024. Demographic and clinical data were collected for these patients, who were stratified by frailty status. We compared these data and investigated factors associated with frailty within this cohort.

**Results:**

The study cohort comprised 160 geriatric inpatients, with 109 (68.1%) classified as frail. Anemia was identified in 65 (40.6%) of the inpatients. The incidence of anemia was significantly higher among frail than among nonfrail geriatric inpatients (*P* = 0.003). Anemia was also significantly correlated with frailty incidence (*P* < 0.001) and with total scores on the Tilburg Frailty Indicator (TFI) (*P* < 0.001), as well as with TFI scores pertaining to the physical (*P* < 0.001) and psychological (*P* = 0.01) dimensions of frailty. Logistic regression identified age (*P* = 0.001), anemia (*P* = 0.047), and lack of regular exercise (*P* = 0.001) as significant risk factors for frailty in this population.

**Conclusions:**

Our study revealed a correlation between anemia and frailty among geriatric inpatients, highlighting the critical need to incorporate vigilant monitoring and management of anemia into the nursing care regimen for these patients.

Frailty, a syndrome common among older adults, refers to the gradual decline in physiological reserve capacity with advancing age, leading to increased vulnerability and a diminished ability to withstand various stressors.^1^ This condition not only elevates health risks among older adults but also profoundly impacts their quality of life. In the hospitalized geriatric population, the prevalence of frailty is particularly notable.^2^,^3^ Guo and colleagues found that the incidence of frailty among hospitalized older adults is as high as 34.7%.^4^ A meta-analysis reported similar data, indicating that the prevalence of frailty among hospitalized older patients is 26.8%.^5^

Frailty is not only a widespread problem but is also closely associated with multiple adverse health outcomes. Research indicates that frail older adults are more susceptible to serious health issues such as falls, disability, cognitive dysfunction, delirium, and even death.^6^ These risks not only pose a threat to the health of older patients but also impose a significant economic burden on families and society.^7^,^8^ Frailty among hospitalized older adults also poses challenges to nurses given its prevalence. It is critical that nurses identify and manage frailty. They must have a thorough understanding of it and a familiarity with effective strategies to address it. Given the far-reaching consequences of frailty, which impact not only the patient's well-being but also families and society, it is imperative that nursing interventions are evidence-based and tailored to enhance patient care and outcomes.

Like frailty, anemia is commonly found among hospitalized older patients. Studies show that the incidence of anemia in this population is as high as 41.02%.^9^,^10^ Anemia in older adults may be due to factors such as decreased immunity, reduced organ function, and poor nutritional status. It often occurs subtly and slowly, making it difficult to detect promptly.^11^,^12^ Some studies have noted that even mild anemia can be an independent risk factor for illness and frailty in older adults.^13^ This association could have profound effects on the health status of this vulnerable population.

**Study purpose**. This study aims to explore the relationship between anemia and frailty in geriatric hospitalized patients in order to establish a more robust foundation for identifying and managing frailty and mitigating its progression.

## METHODS

**Study design**. This study used a retrospective cohort design. It was conducted with the approval of the ethics committee of the First Affiliated Hospital of Soochow University, Suzhou, Jiangsu, China. Written informed consent was obtained from all participants. The data collected were used exclusively for the purposes of the study, and all relevant information was anonymized to ensure participant confidentiality.

The study enrolled patients admitted to the geriatric department of our hospital between October 2022 and August 2024. The inclusion criteria were as follows: (1) being 65 years of age or older; (2) having clear consciousness and the capability to read and verbally communicate; and (3) demonstrating comprehension of the study's objectives and procedures, which, along with the voluntary provision of informed consent, signified an active desire to participate in the research. Patients were excluded if they had severe physical conditions, cognitive deficits, or psychiatric disorders.

The American Geriatrics Society and organizations like One Health Trust (formerly the Center for Disease Dynamics, Economics and Policy) use the designation “older adult” to categorize individuals who have reached the age of 65 years.^14^ This terminology is used to delineate a specific demographic cohort in this study, recognizing the unique health, social, and economic considerations pertinent to this age group. In this study, we used the Tilburg Frailty Indicator (TFI) to assess the frailty status of older patients.^15^ This tool takes into account three critical dimensions of frailty: physical, psychological, and social, which encompass a total of 15 assessment items.^16^ The scale ranges from 0 to 15 points, with the physical dimension scored from 0 to 8, the psychological dimension from 0 to 4, and the social dimension from 0 to 3. According to the TFI scoring criteria, a total score of 5 or above indicates frailty, with higher scores indicating a more severe degree of frailty.^15^ Tests show that the scale has good reliability and validity (Cronbach α = 0.71) and can effectively assess the frailty status of older adults.^17^,^18^

We collected the following data from the participants' medical record: gender, age, height, body weight, body mass index (BMI), systolic blood pressure, diastolic blood pressure, heart rate, hemoglobin level, education level, marital status, residential status, health care payment, sleep quality, number of hospital stays, number of falls, number of comorbidities, number of medications taken, smoking status, alcohol use, and regular exercise. Based on established diagnostic criteria for anemia from the World Health Organization, we set gender-specific hemoglobin thresholds: a concentration below 130 g/L for men and a concentration below 120 g/L for nonpregnant adult women.^19^,^20^

**Data analysis**. In this study, we conducted data analysis using IBM SPSS 24 statistical software. Quantitative data were presented as mean (standard deviation) or median (interquartile range), while qualitative data were expressed as frequencies and percentages. For comparing two groups of quantitative data, we used either the independent samples *t* test or the Mann-Whitney *U* test, depending on the distribution characteristics of the data. For the comparison of qualitative data between groups, we used the chi-square (χ^2^) test or Fisher exact test. We also used Spearman correlation analysis to explore the relationship between frailty and anemia. To identify risk factors associated with frailty, we conducted binary logistic regression analysis. In all tests, *P* < 0.05 was considered statistically significant.

## RESULTS

A total of 160 geriatric inpatients were enrolled, of whom 109 were identified as frail, for a frailty incidence of 68.1%. This study found significant differences between nonfrail and frail geriatric inpatients in terms of age, weight, BMI, heart rate, hemoglobin level, marital status, residential status, number of hospital stays, number of medications, alcohol use, and regular exercise habits. Specifically, compared with nonfrail geriatric inpatients, frail inpatients were older and had lower body weight, lower BMI, slower heart rate, lower hemoglobin levels, and a higher incidence of being widowed. A greater proportion of frail inpatients were living alone or with children, had three to four or more than six hospitalizations, and were taking more than five medications; a smaller proportion engaged in regular exercise. See Table 1 for more details.

**Table 1.** Demographic Characteristics of Geriatric Inpatients (N = 160)

Variables	No Frailty (n = 51)	Frailty (n = 109)	*t*/χ^2^	*P*
Gender, n (%)			2.401	0.12
Male	39 (76.5)	70 (64.2)		
Female	12 (23.5)	39 (35.8)		
Age in years, mean (SD)	79.35 (9.30)	87.20 (6.07)	−5.746	< 0.001
Height, cm, mean (SD)	165.00 (7.20)	163.01 (8.81)	−1.114	0.27
Body weight, kg, mean (SD)	66.10 (6.83)	62.32 (9.80)	−2.286	0.02
BMI, mean (SD)^a^	24.28 (2.10)	23.32 (2.79)	2.435	0.02
Systolic blood pressure, mmHg, mean (SD)	133.61 (15.21)	135.19 (19.97)	0.539	0.59
Diastolic blood pressure, mmHg, mean (SD)	71.39 (10.51)	70.40 (10.94)	−0.614	0.54
Heart rate, beats per minute, mean (SD)	78.16 (10.88)	75.78 (12.00)	−1.967	0.049
Hemoglobin, g/L, mean (SD)	130.10 (18.65)	114.32 (18.95)	4.902	< 0.001
Education level, n (%)			4.095	0.39
Primary school	0 (0.0)	2 (1.8)		
Junior high school	18 (35.3)	27 (24.8)		
Senior high school	8 (15.7)	20 (18.3)		
Vocational college	15 (29.4)	44 (40.4)		
University	10 (19.6)	16 (14.7)		
Marital status, n (%)			14.894	< 0.001
Married	44 (86.3)	60 (55)		
Widowed	7 (13.7)	49 (45)		
Residential status, n (%)			15.268	< 0.001
Living alone	5 (9.8)	20 (18.3)		
Living with spouse	43 (84.3)	58 (53.2)		
Living with children	3 (5.9)	31 (28.4)		
Health care payment, n (%)			0.009	0.92
Out of pocket	3 (5.9)	6 (5.5)		
Health insurance	48 (94.1)	103 (94.5)		
Sleep quality, n (%)			7.971	0.09
Very good	1 (2)	1 (0.9)		
Good	18 (35.3)	18 (16.5)		
Average	25 (49)	66 (60.6)		
Poor	6 (11.8)	22 (20.2)		
Very poor	1 (2)	2 (1.8)		
No. of hospital stays, n (%)			10.089	0.02
1-2	46 (90.2)	81 (74.3)		
3-4	2 (3.9)	12 (11)		
5-6	3 (5.9)	3 (2.8)		
> 6	0 (0.0)	13 (11.9)		
No. of falls, n (%)			4.193	0.12
0	47 (92.2)	94 (86.2)		
1-2	3 (5.9)	15 (13.8)		
≥ 3	1 (2)	0 (0.0)		
No. of comorbidities, n (%)			1.911	0.39
0	5 (9.8)	6 (5.5)		
1-2	43 (84.3)	91 (83.5)		
3-4	3 (5.9)	12 (11)		
No. of medications, n (%)			9.193	0.04
0	1 (2)	4 (3.7)		
1-2	15 (29.4)	14 (12.8)		
3-4	28 (54.9)	57 (52.3)		
5-6	5 (9.8)	23 (21.1)		
> 6	2 (3.9)	11 (10.1)		
Smoking status, n (%)			5.866	0.12
Never	31 (60.8)	72 (66.1)		
Occasional	4 (7.8)	1 (0.9)		
Frequent	1 (2)	1 (0.9)		
Former	15 (29.4)	35 (32.1)		
Alcohol use, n (%)			13.307	0.004
Never	27 (52.9)	65 (59.6)		
Occasional	13 (25.5)	8 (7.3)		
Frequent	2 (3.9)	1 (0.9)		
Former	9 (17.6)	35 (32.1)		
Regular exercise, n (%)			23.958	< 0.001
No	8 (15.7)	62 (56.9)		
Yes	43 (84.3)	12 (43.1)		

BMI = body mass index.

a Calculated as weight in kilograms divided by height in meters squared.

Note: Percentages may not sum to 100 due to rounding.

Among the geriatric inpatients enrolled in the study, there were 65 cases of anemia for an incidence of 40.6%. The incidence of anemia was significantly higher among frail older inpatients than among nonfrail older inpatients (χ^2^ = 9.07, *P* = 0.003). Furthermore, as shown in Table 2, the study revealed a positive correlation between anemia and frailty (*r* = 0.24, *P* < 0.001).

**Table 2.** Correlation Between Anemia and Frailty in Geriatric Inpatients (N = 160)

Anemia	No Frailty (n = 51) n (%)	Frailty (n = 109) n (%)	*r*	*P*
No	39 (76.5)	56 (51.4)	0.24	< 0.001
Yes	12 (23.5)	53 (48.6)		

Spearman correlation analysis was used to examine the relationship between the TFI scale total score, the subscale scores, and the occurrence of anemia. As seen in Table 3, results indicate a significant correlation between the occurrence of anemia and the total TFI score (*P* < 0.001), as well as the physical (*P* < 0.001) and psychological (*P* = 0.01) dimension scores.

**Table 3.** Correlation Between Anemia and Frailty Scores in Geriatric Inpatients

Scores	*r*	*P*
Total TFI score	0.28	< 0.001
Physical dimension	0.29	< 0.001
Psychological dimension	0.20	0.01
Social dimension	0.08	0.31

TFI = Tilburg Frailty Indicator.

In this study, we incorporated variables with statistically significant results from the univariate analysis into a binary logistic regression model to identify the factors influencing the occurrence of frailty among geriatric inpatients. As shown in Table 4, through this analysis, we identified age, anemia status, and lack of regular exercise habits as significant factors affecting frailty.

**Table 4.** Logistic Analysis of Factors Influencing Frailty in Geriatric Inpatients

Variable	Regression Coefficient	Standard Error	Wald Test	*P*	Standardized Regression Coefficient	95% CI
Age	0.12	0.04	10.32	0.001	1.13	1.05-1.22
Anemia	−1.06	0.53	3.95	0.047	0.35	0.12-0.99
Lack of regular exercise	2.29	0.68	11.38	0.001	9.90	2.61-37.54

## DISCUSSION

With the growth of the older population globally, aging has emerged as a widely concerning public health issue. China, one of the countries with the largest older populations worldwide, is confronting the increasingly severe health challenges posed by an aging demographic. For example, the growing demand for health care services by older adults puts a strain on medical resources, including hospital beds, specialized geriatric care units, and health care professionals trained in geriatrics. Among older adults, anemia and frailty are two prevalent syndromes that reflect deficiencies and imbalances within the body. Anemia refers to a reduction in levels of hemoglobin, which is crucial for oxygen transport. In cases of anemia, the body's capacity to transport oxygen is diminished, potentially leading to organ dysfunction and, if persistent, organ failure. In older patients with anemia, the functional reserve capacity of tissues and organs gradually decreases with age, thereby increasing the risk of frailty.

The findings of this study reveal that the incidence of frailty among geriatric inpatients was 68.1%, a proportion higher than that reported by Wei and colleagues, who found a frailty incidence of 34.4% among hospitalized patients over 65 years of age.^21^ This discrepancy may be due to the use of different assessment tools. Our study used the TFI scale, which evaluates frailty across physiological, psychological, and social dimensions, whereas Wei and colleagues used the Frail Scale, which assesses frailty from a physiological perspective only. Furthermore, the incidence of anemia among geriatric inpatients in our study was 40.6%, a proportion similar to that in previous relevant studies.^10^ These data suggest that both frailty and anemia are highly prevalent among geriatric inpatients, necessitating greater attention paid to these conditions and the implementation of appropriate preventive and interventional measures.

This study showed a correlation between anemia and frailty incidence, and found that anemia is associated with the total TFI score and the physical and psychological dimension scores. This finding is consistent with previous research outcomes.^12^,^22^ However, the connection between anemia and frailty is not fully understood. It may be influenced by inflammation, which is increasingly understood as a core characteristic of the aging process. Current research indicates that in older patients with frailty, inflammatory markers such as interleukins, C-reactive protein, tumor necrosis factor, and white blood cell counts are significantly increased.^23^-^26^ These inflammatory markers may directly or indirectly affect the hematopoietic system, causing structural and functional damage to the body's tissues and organs, thereby promoting the occurrence of frailty. Other studies have confirmed the association between inflammatory markers and oxidative stress.^27^,^28^ Increased oxidative stress exacerbates the disease burden in older adults and contributes to the development of chronic anemia, which may ultimately result in frailty.^29^,^30^ Therefore, inflammation and oxidative stress may be important biological pathways linking anemia to frailty, which warrants further analysis and exploration in future research.

Age, anemia, and inactivity are independent risk factors for the development of frailty among older adults.^31^,^32^ With increasing age, there is a concomitant deterioration in various physiological parameters. This encompasses a gradual decrease in muscular strength, a diminution in sensory perception, and a slowdown in metabolic processes. Collectively, these age-related physiological changes can precipitate a transition toward a state of frailty, which is characterized by an increased vulnerability to adverse health outcomes.^33^,^34^ Furthermore, it is not uncommon for older adults to be afflicted with multiple chronic conditions. The presence of these comorbidities, coupled with the complex therapeutic regimens often required to manage them, can exacerbate the trajectory of frailty. This exacerbation is attributed to the cumulative burden of disease and treatment-related side effects, which may impose additional stress on the already compromised physiological reserves of older adults, thereby hastening the onset and progression of frailty.^35^

Anemia, defined by a lower than normal level of red blood cells or hemoglobin in the blood, leads to a decreased ability to transport oxygen to various parts of the body.^36^,^37^ Older adults are more susceptible to anemia due to reduced digestive function, inadequate nutrient absorption, or chronic diseases.^38^ Anemia not only causes fatigue, weakness, and cognitive decline but can also exacerbate the symptoms of frailty in older adults.^39^

Older individuals who lack regular exercise are more prone to muscle atrophy, decreased bone density, and physical decline, all of which are potential manifestations of frailty.^40^,^41^ Therefore, encouraging older adults to engage in appropriate physical activities—and supporting their engagement—is important for the prevention and delay of frailty.^42^

**Implications for nursing practice**. In response to the identified risk factors for the development of frailty, it is essential to implement a suite of comprehensive, evidence-based nursing care measures that cater to the unique needs of the geriatric population. These interventions are designed to enhance the quality of life for older adults and reduce the incidence of frailty. Health education plays a pivotal role in empowering older adults to make informed health decisions, including understanding the importance of early detection and management of anemia, maintaining a balanced diet, and engaging in regular physical activity. Nurses are instrumental in guiding patients through these steps and emphasizing the importance of regular health checkups.

Nutritional support is a critical component of care, given the physiological changes associated with aging. Nurses should assess and address the nutritional status of older patients, offering dietary advice and, when necessary, oral nutrition supplements to prevent undernutrition.^43^ The administration of iron supplements is one such intervention that can directly impact the management of anemia, a condition shown to be closely linked to frailty. This personalized approach to nutrition is crucial and often involves coordination with dietitians to develop meal plans that meet the specific dietary needs of each patient.

Regular exercise is an effective means of preventing frailty. Exercise can enhance muscle strength, improve cardiopulmonary function, and ameliorate balance and coordination, thereby reducing frailty risk.^44^,^45^ Physical activity is a cornerstone of improving functional capacity. Nurses should assess each patient's physical capabilities and work with physiotherapists to design personalized exercise programs that may include balance training, resistance exercises, and aerobic activities tailored to individual preferences and abilities. Fall prevention strategies are also integral to reducing the risk of injury and disability in older adults. Nurses can help implement these strategies, which may include environmental modifications, gait and balance training, and the use of assistive devices, to mitigate the risk of falls and subsequent frailty.^43^

Interdisciplinary collaboration is vital to address the multifaceted needs of older patients. Nurses should collaborate with physicians, social workers, and community resources to ensure a holistic approach to care that encompasses physical health as well as psychological, social, and cultural factors contributing to frailty.

**Limitations**. The present study has several limitations that may impact the interpretation of the results. Because it was conducted in a single center and had a relatively small sample size, the conclusions drawn may not be fully representative of a broader population or different geographical regions, introducing potential biases related to the specific population or area. In addition, the retrospective study design limited the scope of the factors associated with frailty that could be collected and analyzed, and there may be other factors not included in this analysis that could influence the frailty status of older adults. Lastly, while this study demonstrated a correlation between anemia and frailty, the causal relationship between the two remains unclear. It is not yet understood whether anemia leads to frailty, if frailty increases the risk of anemia, or if the two influence each other through other mechanisms. Further in-depth exploration and validation are needed in future research. Therefore, the findings of this study should be interpreted with caution and substantiated in subsequent studies.

## CONCLUSION

Our study revealed that the incidence of both frailty and anemia was notably high among geriatric inpatients. Furthermore, a significant correlation exists between anemia and frailty in this patient population, suggesting that anemia is a crucial factor in the development of frailty. These findings underscore the importance of recognizing and addressing anemia as a potentially modifiable risk factor in the context of frailty management. The co-occurrence of these two conditions highlights the need for a more integrated and comprehensive approach to the care of older patients. Nursing interventions can play a critical role in addressing the complex health needs of frail older patients. Coordination of care with hematologists is essential for comprehensive treatment plans, as these specialists can provide insights into the underlying causes of anemia and contribute to the development of targeted nursing care measures.
